# Vein of Marshall Collateralization during Ethanol Infusion in Atrial Fibrillation: Solution for Effective Myocardium Staining

**DOI:** 10.3390/jcm12010309

**Published:** 2022-12-30

**Authors:** Hongda Zhang, Lei Ding, Lijie Mi, Kuo Zhang, Zihan Jiang, Sixian Weng, Fengyuan Yu, Min Tang

**Affiliations:** 1 Arrhythmia Center, State Key Laboratory of Cardiovascular Disease, Fuwai Hospital, National Center for Cardiovascular Diseases, Chinese Academy of Medical Sciences & Peking Union Medical College, Beijing 100037, China; 2 National Center for Clinical Laboratories, Institute of Geriatric Medicine, Chinese Academy of Medical Sciences, Beijing Hospital, National Center of Gerontology, Beijing 100037, China

**Keywords:** atrial fibrillation, vein of Marshall, ethanol infusion, collateral circulation, slow injection

## Abstract

Background: The vein of Marshall (VOM) ethanol infusion improves sinus rhythm maintenance in patients with atrial fibrillation (AF). Distal collateral circulation of VOM can be a challenge to effective ethanol infusion. Objective: This study aimed to evaluate the feasibility and efficacy of ethanol infusion in VOM with distal collateral circulation. Methods: Patients with AF scheduled for catheter ablation and VOM ethanol infusion were consecutively enrolled. During the procedure, non-occluded coronary sinus angiography was first performed for VOM identification. After VOM identification, an over-the-wire angioplasty balloon was used for cannulation and occluded angiography of the VOM. Those with distal VOM collateral circulation were included in this study. A method of slower ethanol injection (2 mL over 5 min) plus additional balloon occlusion time for 3 min after each injection was used. Results: Of 162 patients scheduled for VOM ethanol infusion, apparent distal VOM collateral circulation was revealed in seven (4.3%) patients. Five patients had collateral circulation to the left atrium, one to the right superior vena cava, and one to the great cardiac vein. Two patients did not undergo further ethanol infusion because of our inadequate experience during the early stage of the project. Five patients had successful VOM ethanol infusion with manifest localized myocardium staining. Conclusions: Ethanol infusion in VOM with distal collateral circulation can be solved by slow injection of ethanol and enough balloon occlusion time between multiple injections.

## 1. Introduction

The vein of Marshall (VOM), a remnant of the left superior vena cava, has been proven to be an arrhythmogenic source in patients with atrial fibrillation (AF) [1,2,3,4]. VOM ethanol infusion, in combination with catheter ablation of the pulmonary veins, improves sinus rhythm maintenance in patients with persistent AF [1,2,5]. The VOM can be extremely narrow, and its anatomical variability is substantial [2,6,7,8]. Identification and cannulation of the VOM require knowledge of fluoroscopic anatomy and different angioplasty tools. VOM ethanol infusion can be technically challenging. There has been a growing body of literature focusing on techniques of VOM ethanol infusion [7,9,10]. 

Prior studies have reported that VOM can have none or many branches, be a venous plexus, and have collateral circulation to the left atrium, great cardiac vein, coronary sinus, or superior vena cava [2,8]. Collateral circulation of VOM can be a challenge to effective ethanol infusion. In centers with a low volume of VOM ethanol infusion, collateral circulation might prevent inexperienced operators from further ethanol infusion. In cases of ethanol infusion attempts, collateral circulation might weaken the effect of VOM ethanol infusion. This study aimed to evaluate the feasibility and efficacy of ethanol infusion in VOM with distal collateral circulation.

## 2. Methods

### 2.1. Patient Selection

Patients with AF who underwent radiofrequency catheter ablation and VOM ethanol infusion in Fuwai Hospital, Beijing, China, between November 2021 and September 2022 were consecutively enrolled. Those with distal VOM collateral circulation were further included in this study. This study was performed in accordance with the Declaration of Helsinki and was approved by the Ethics Committee of Fuwai Hospital. Informed consent was obtained from all participants. 

### 2.2. Procedural Approach

The procedure included pulmonary vein isolation, coronary sinus angiography for VOM identification, VOM cannulation and ethanol infusion, and catheter ablation of the mitral isthmus. The technical details of catheter ablation have been previously reported [7].

After catheter ablation, a guiding catheter (6-F Judkins Right [JR] 4; Medtronic, Minneapolis, MN, USA) was positioned inside the coronary sinus (CS) through an SL1 long sheath (8.5F; St. Jude Medical, Inc., St Paul, MN, USA) or a flexible long sheath (8.5-F, Agilis NxT; Abbott, St Paul, MN, USA) inserted from the right femoral vein. Non-occluded coronary sinus angiography was performed in the right anterior oblique (RAO), the left anterior oblique (LAO), and the LAO cranial views for VOM identification [7]. After VOM identification, an over-the-wire angioplasty balloon positioned in distal VOM was used for cannulation and occluded angiography of the VOM. If there was no distal VOM collateral circulation in either the non-occluded coronary sinus angiography or occluded VOM angiography, ethanol was delivered slowly at 2–3 positions distally to proximally in the VOM. After ethanol infusion, catheter ablation of the mitral isthmus was performed. If there was apparent distal VOM collateral circulation, a method of slower ethanol injection (2 mL over 5 min) plus additional balloon occlusion time for 3 min after each injection was used. Manifest localized myocardium staining was considered successful ethanol infusion.

### 2.3. Statistical Analysis

Continuous variables are expressed as mean ± standard deviation or median (interquartile range) as appropriate, and categorical variables are shown as ratio or percentage. Data analyses were performed using R software, version 4.2.0 (R Core Team, Vienna, Austria).

## 3. Results

### 3.1. Baseline Characteristics

A total of 162 patients were enrolled, and apparent distal VOM collateral circulation was found in 7 (4.3%) patients. The baseline characteristics of these seven patients are displayed in Table 1. They all had persistent AF. Patients had a mean age of 59 ± 7 years old, and 3 (28.6%) were female. The mean left atrium dimension was 43 ± 2.0 mm. 

### 3.2. Procedural Details

All seven patients had successful VOM cannulation. Five patients had distal collateral circulation to the left atrium, one to the right superior vena cava, and one to the great cardiac vein. Two patients (Patients No. 1–2) did not undergo further ethanol infusion because of our inadequate experience during the early stage of the project. Five patients (Patients No. 3–7) had successful VOM ethanol infusion with manifest localized myocardium staining. 

### 3.3. Examples of VOM Collateralization without Ethanol Infusion Attempt

**Figure 1:**
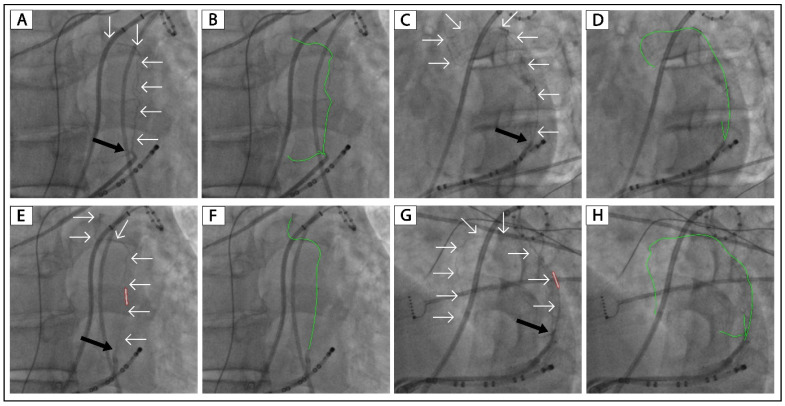
VOM collateralization in a patient without ethanol infusion attempt. (**A**,**C**): Non-occluded venograms of VOM in the RAO and LAO projection, respectively. (**E**,**G**): Balloon occluded venograms of VOM in the RAO and LAO projection, respectively. (**B**,**D**,**F**,**H**): Highlighted VOMs of panels (**A**,**C**,**E**,**G**), respectively. The black arrow indicates the ostium of VOM. The white arrows and green lines indicate the drainage of the VOM. The tubule outlined in red indicates the angioplasty balloon. VOM, the vein of Marshall; RAO, right anterior oblique; LAO, left anterior oblique.

Figure 1 shows a patient (Patient No. 2) with VOM collateral circulation to the left atrium. The collateral circulation was first revealed in non-occluded VOM venograms in the RAO and LAO projection. Further balloon-occluded VOM venograms confirmed the existence of collateral circulation. Supplemental Figure S1 (Patient No. 1) showed another patient with VOM collateral circulation to the left atrium. In these two patients, ethanol infusion was not performed. 

### 3.4. Examples of VOM Collateralization with Successful Ethanol Infusion

**Figure 2:**
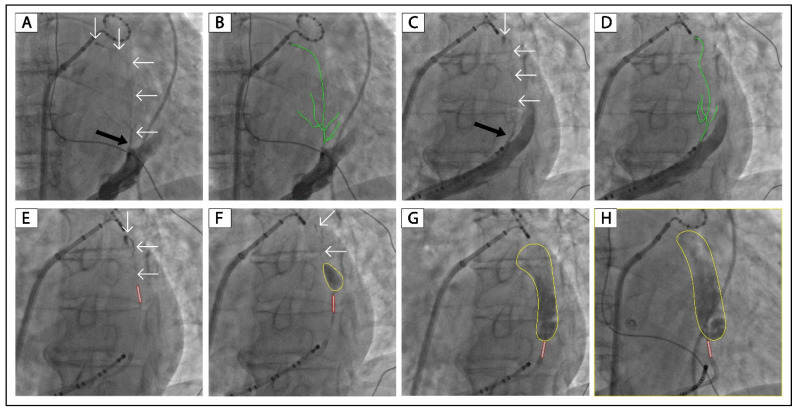
Successful ethanol infusion in a VOM with collateral circulation to the left atrium. (**A**,**C**): Non-occluded venograms of VOM in the RAO and LAO projection, respectively. (**B**,**D**): Highlighted VOMs of panels (**A**,**C**), respectively. (**E**–**G**): Balloon occluded venograms of VOM in the LAO projection after 2, 4, and 8 mL of ethanol infusion, respectively. (**H**): Balloon occluded venogram of VOM in the RAO projection after 8 mL of ethanol infusion. The black arrow indicates the ostium of VOM. The white arrows and green lines indicate the drainage of the VOM. The tubule outlined in red indicates the angioplasty balloon. The yellow circles indicate localized myocardium staining. VOM, the vein of Marshall; RAO, right anterior oblique; LAO, left anterior oblique.

Figure 2 displays another patient (Patient No. 3) with VOM collateral circulation to the left atrium. After a slow injection of 2 mL of ethanol, there was still collateral circulation and no localized staining (Figure 2E). After 4 mL of ethanol infusion, there was lesser collateral circulation than in Figure 2E and a small area of localized staining (Figure 2F). After 8 mL of ethanol infusion, there was no collateral circulation, and the size of localized staining was more prominent, as shown in the LAO and RAO projections (Figure 2G,H). Supplemental Figures S2 and S3 show another two patients with VOM collateral circulation to the left atrium in whom ethanol infusion was successful. 

**Figure 3:**
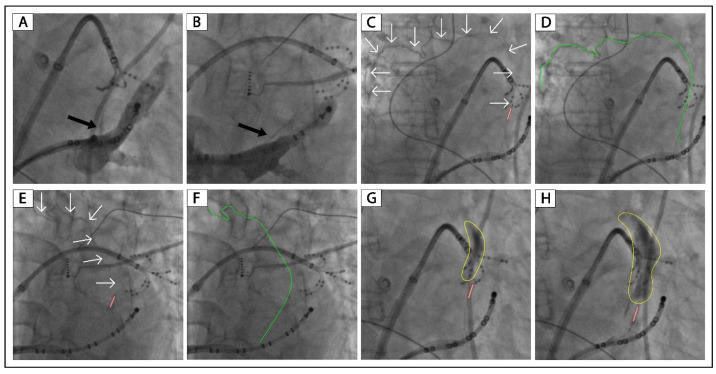
Successful ethanol infusion in a VOM with collateral circulation to the right superior vena cava. (**A**,**B**): Non-occluded venograms of VOM in the RAO and LAO projection, respectively. C and E: Balloon occluded venograms of VOM in the RAO and LAO projection, respectively. (**D**,**F**): Highlighted VOM of panels (**C**,**E**). (**G**,**H**): Balloon occluded venograms of VOM in the RAO projection after 4 and 8 mL of ethanol infusion, respectively. The black arrow indicates the ostium of VOM. The white arrows and green lines indicate the drainage of the VOM. The tubule outlined in red indicates the angioplasty balloon. The yellow circles indicate localized myocardium staining. VOM, the vein of Marshall; RAO, right anterior oblique; LAO, left anterior oblique.

**Figure 4:**
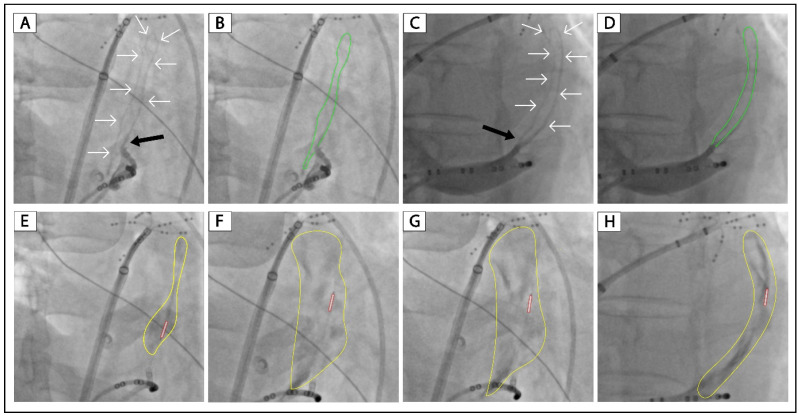
Successful ethanol infusion in a VOM with collateral circulation to the great cardiac vein. (**A**,**C**): Non-occluded venograms of VOM in the RAO and LAO projection, respectively. (**B**,**D**): Highlighted VOMs of panels (**A**,**C**), respectively. (**E**–**G**): Balloon occluded venograms of VOM in the RAO projection after 2, 4, and 8 mL of ethanol infusion, respectively. (**H**): Balloon occluded venograms of VOM in the LAO projection after 8 mL of ethanol infusion. The black arrow indicates the ostium of VOM. The white arrows and green lines indicate the drainage of the VOM. The tubule outlined in red indicates the angioplasty balloon. The yellow circles indicate localized myocardium staining. VOM, the vein of Marshall; RAO, right anterior oblique; LAO, left anterior oblique.

Figure 3 shows a patient (Patient No. 4) with VOM collateral circulation to the right superior vena cava. After 8 mL of ethanol infusion, there was no collateral circulation, and a large area of localized staining was presented (Figure 3H). Figure 4 shows a patient (Patient No. 5) with VOM collateral circulation to the great cardiac vein. After 8 mL of ethanol infusion, there was no collateral circulation, and a large area of localized staining was shown (Figure 4G,H). 

**Figure 5:**
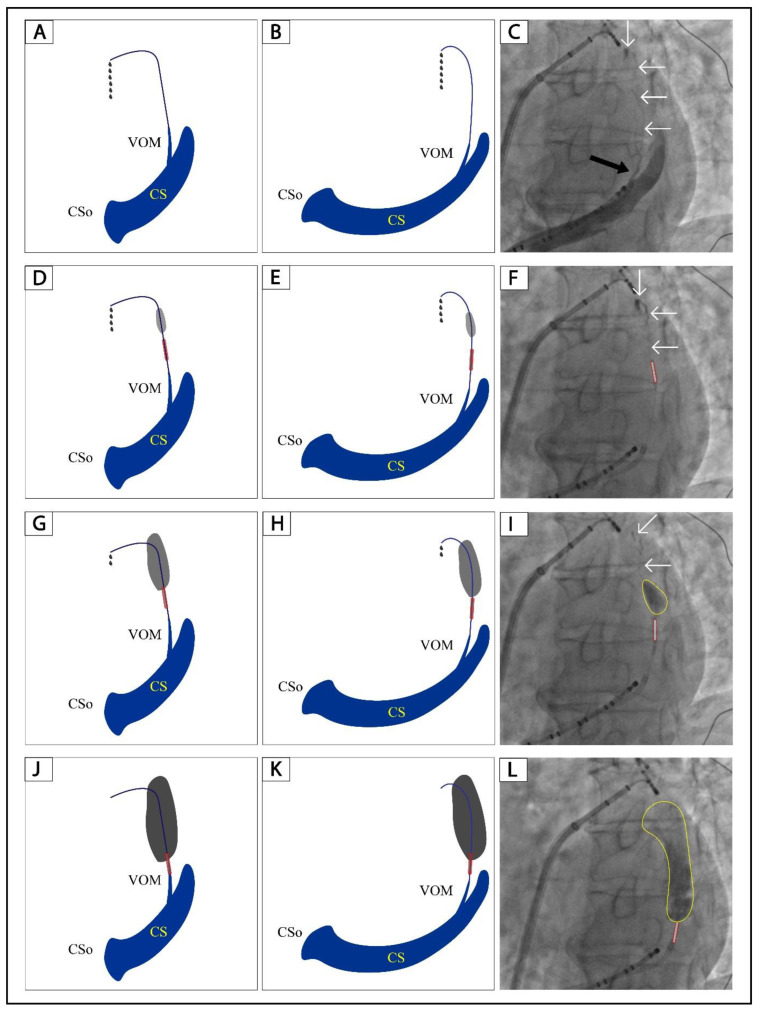
A step-by-step illustration of ethanol infusion in a VOM with collateral circulation. (**A**,**B**): Schematic diagram of non-occluded venograms of VOM in the RAO and LAO projection, respectively. (**D**,**G**,**J**): Schematic diagram of balloon occluded venograms of VOM in the RAO projection after 2, 4, and 8 mL of ethanol infusion, respectively. (**E**,**H**,**K**): Schematic diagram of balloon occluded venograms of VOM in the LAO projection after 2, 4, and 8 mL of ethanol infusion, respectively. (**C**): Non-occluded venogram of VOM in the LAO projection. (**F**,**I**,**L**): Balloon occluded venograms of VOM in the RAO projection after 2, 4, and 8 mL of ethanol infusion, respectively. The black arrow indicates the ostium of VOM. The white arrows indicate the drainage of the VOM. The tubule outlined in red indicates the angioplasty balloon. The gray and black shadows in Panels (**D**,**E**,**G**,**H**,**J**) and yellow circles in Panels (**I**,**L**) indicate localized myocardium staining. The black dots in Panels (**A**,**B**,**D**,**E**,**G**,**H**) indicate contrast leakage through the collateral circulation. VOM, the vein of Marshall; RAO, right anterior oblique; LAO, left anterior oblique.

The solution to VOM collateral circulation during ethanol infusion is shown in Figure 5. VOM collateral circulation can be revealed by non-occluded coronary sinus angiography and occluded VOM angiography (Figure 5A–F). After 2–6 mL of ethanol infusion as slowly as possible, the collateral circulation will be reduced or disappear (Figure 5D–I). There will be no collateral circulation after 6–8 mL of ethanol circulation, and a large area of localized staining will be presented (Figure 5J–L). The voltage maps of the left atrial lateral wall following ethanol administration are displayed in Supplemental Figure S4. Of the five patients with ethanol infusion, three had no atrial arrhythmias over a 3-month follow-up, and two had no atrial arrhythmias over a 6-month follow-up. No adverse events occurred during the follow-up.

## 4. Discussion

This study introduces our experience in ethanol infusion in VOM with distal collateral circulation. The results showed that VOM could connect with adjacent vessels or chambers distally and that slow ethanol injection could occlude the anastomosis and facilitate successfully localized myocardium staining. 

In this study, only 4.9% of all patients had collateral circulation, which was significantly lower than in previous studies [2]. The main reason for this might be the different angiography methods used in our center, which only identified distal VOM collateral circulation. The first is non-occluded angiography for VOM identification. As we conclude, the key to identifying the VOM is to perform angiography in three fluoroscopic views and at least three positions of the guiding catheter in the coronary sinus lumen from distal to proximal. A balloon occlusion for coronary sinus angiogram is no longer needed in our lab, simplifying the procedure and shortening the procedure time. The second is the different locations of the balloon for occluded VOM angiography. We always advance the balloon as distal as possible rather than in the ostium in the VOM to perform VOM angiography and the first ethanol injection. So, a large proportion of collateral circulation originating from the proximal-to-medium VOM might be missing in our study. Similar to previous studies, the distal collateral connections included the left atrium, great cardiac vein, coronary sinus, and superior vena cava [2,8]. 

In patients No. 1–2, ethanol infusion was not performed because of our inadequate experience in the early stage of the VOM ethanol infusion project. As shown in Figure 1 and Supplemental Figure S1, these two patients had VOM collateral circulation to the left atrium, like the successful case shown in Figure 2. We believe ethanol infusion could occlude the connections between the distal VOM and the left atrium.

Figure 2, Figure 3 and Figure 4 and Supplemental Figures S2 and S3 show the successful cases of ethanol infusion with manifest localized staining (Patients No. 3–7). Three had collateral circulation to the left atrium, one to the right superior vena cava, and one to the great cardiac vein. We conclude that the key to successful ethanol infusion in VOM with collateral circulation is the injection speed and the waiting time between multiple injections. In regular cases, we inject 2 mL of ethanol over 1 min and wait 1 min for the next injection. In such cases with collateral circulation, we will increase the injection time to 5 min for 2 mL of ethanol. And the waiting time between injections will be increased to 3 min. The anastomosis between distal VOM and adjacent vessels or chambers could be occluded using this method. The underlying reason might be that the anastomosis is always narrow, and the blood flow through it is slow. We succeeded in all five patients with VOM collateral circulation using this method. 

## 5. Limitations

This study was conducted in a single center with only seven cases with VOM collateral circulation. The rate of VOM collateral circulation was low in our study because of the different methods used for angiography. We only identified distal VOM collateral circulation, which was crucial to ethanol infusion. Multicenter prospective studies with larger sample sizes are warranted in the future.

## 6. Conclusions

Ethanol infusion in VOM with collateral circulation can be solved by slow injection of ethanol and enough balloon occlusion time between multiple injections.

## Figures and Tables

**Table 1:** Patient characteristics and procedural data.

Patient No.	Age, y	Sex	BMI, kg/m^2^	Comorbidities	CHA_2_DS_2_-VASc Score	HAS-BLED Score	LA, mm	LVEF,%	Cannulation Success	Ethanol Infusion Success
1	58	Male	23.4	HTN, DM, stroke	4	1	47	68	Yes	No
2	59	Male	23.9	stroke	2	1	41	65	Yes	No
3	65	Male	25.3	HTN, stroke	4	3	43	63	Yes	Yes
4	59	Female	25.4	HTN, CAD	2	1	43	66	Yes	Yes
5	49	Male	29.3	DM, CAD, PDA	2	0	45	51	Yes	Yes
6	71	Female	22.1		2	1	41	70	Yes	Yes
7	54	Male	26.6	HTN, DM	2	0	43	60	Yes	Yes

BMI, body mass index; HTN, hypertension; CAD, coronary artery disease; DM, diabetes mellitus; PDA, peripheral arterial disease; CHA_2_DS_2_-VASc: congestive heart failure, hypertension, age ≥ 75 years, diabetes mellitus, stroke, vascular disease, age 65–74 years, sex category (female); HAS-BLED: hypertension, abnormal renal/liver function, stroke, bleeding history or predisposition, labile international normalized ratio, elderly (>65 years of age), concomitant drugs/alcohol; LA, left atrium; LVEF, left ventricular ejection fraction.

## Data Availability

Research data is confidential. Data-sharing requests are required to meet the policies of the hospital and the funder.

## Supplementary Materials

The following supporting information can be downloaded at: https://www.mdpi.com/article/10.3390/jcm12010309/s1, Figure S1: VOM collateralization in a patient (Patient No. 1) without ethanol infusion attempt; Figure S2: Successful ethanol infusion in a VOM (Patient No. 6) with collateral circulation to the left atrium; Figure S3: Successful ethanol infusion in a VOM (Patient No. 7) with collateral circulation to the left atrium; Figure S4: Voltage maps of the left atrial lateral wall after ethanol infusion.

## Abbreviations

AF	atrial fibrillation
VOM	vein of Marshall
RAO	the right anterior oblique
LAO	the left anterior oblique
