# Supplementary material for: Vein of Marshall Collateralization during Ethanol Infusion in Atrial Fibrillation: Solution for Effective Myocardium Staining
Source: J Clin Med. 2022 Dec 30;12(1):309. doi: 10.3390/jcm12010309 (PMC9821427; doi:10.3390/jcm12010309)
Supplement: Supplementary file 1 [file jcm-12-00309-s001.zip › jcm-2083406-supplementary.pdf]

Supplemental Figures

Supplemental Figure S1. VOM collateralization in a patient (Patient No. 1) without ethanol infusion attempt.

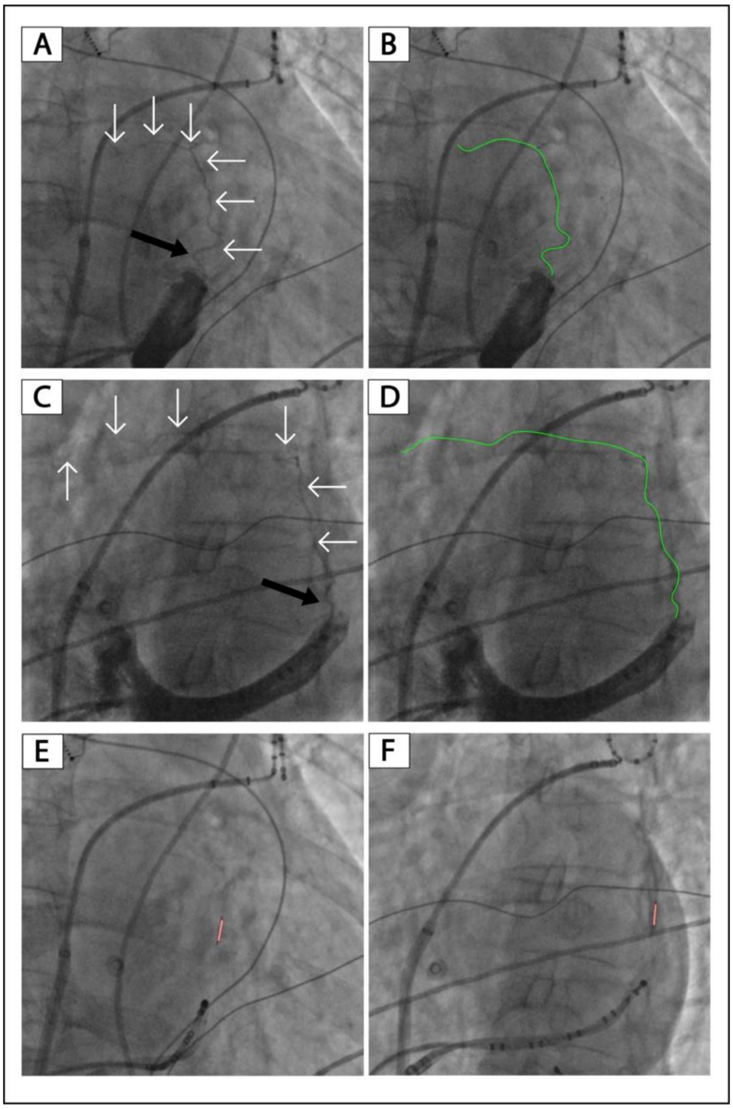

A and C: Non-occluded venograms of VOM in the RAO and LAO projection, respectively. E and F: Balloon occluded venograms of VOM in the RAO and LAO projection, respectively. B, D: Highlighted VOMs of panels A and C, respectively.

The black arrow indicates the ostium of VOM. The white arrows and green lines indicate the drainage of the VOM. The tubule outlined in red indicates the angioplasty balloon.

VOM, the vein of Marshall; RAO, right anterior oblique; LAO, left anterior oblique.

Supplemental Figure S2. Successful ethanol infusion in a VOM (Patient No. 6) with collateral circulation to the left atrium.

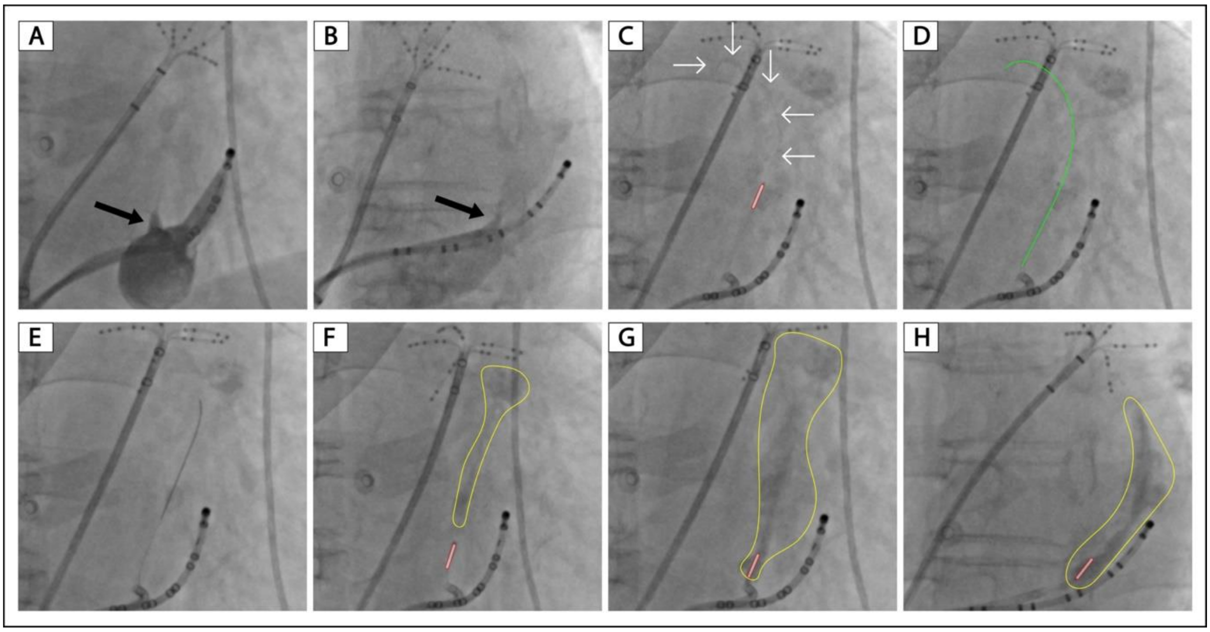

A and B: Non-occluded venograms of VOM in the RAO and LAO projection, respectively. C: Balloon occluded venogram of VOM in the RAO projection. D: Highlighted VOM of panels C. E: Angioplasty wire in the VOM. F and G: Balloon occluded venograms of VOM in the RAO projection after 4 and 8 ml of ethanol infusion, respectively. H: Balloon occluded venogram of VOM in the LAO projection after 8 ml of ethanol infusion.

The black arrow indicates the ostium of VOM. The white arrows and green lines indicate the drainage of the VOM. The tubule outlined in red indicates the angioplasty balloon. The yellow circles indicate localized myocardium staining.

VOM, the vein of Marshall; RAO, right anterior oblique; LAO, left anterior oblique.

Supplemental Figure S3. Successful ethanol infusion in a VOM (Patient No. 7) with collateral circulation to the left atrium.

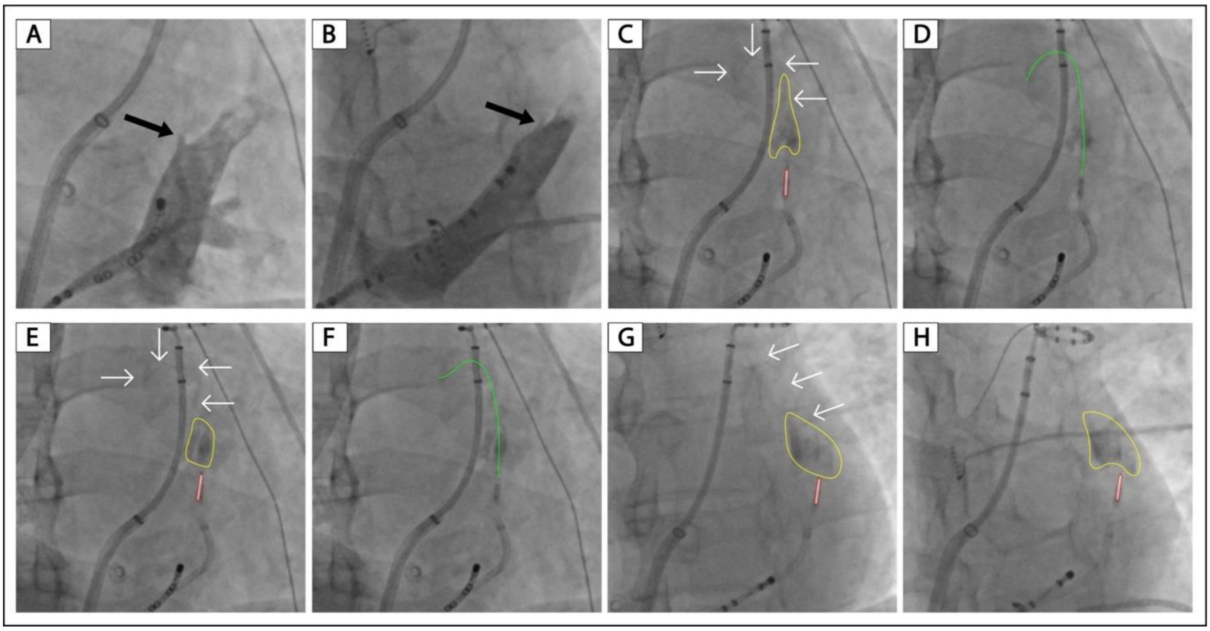

A and B: Non-occluded venograms of VOM in the RAO and LAO projection, respectively. C, E, G, and H: Balloon occluded venograms of VOM in the RAO projection after 2, 4, 6, and 8 ml of ethanol infusion, respectively. D and F: Highlighted VOMs of panels C and E, respectively.

The black arrow indicates the ostium of VOM. The white arrows and green lines indicate the drainage of the VOM. The tubule outlined in red indicates the angioplasty balloon. The yellow circles indicate localized myocardium staining.

VOM, the vein of Marshall; RAO, right anterior oblique; LAO, left anterior oblique.

Supplemental Figure S4. Voltage maps of the left atrial lateral wall after ethanol infusion.

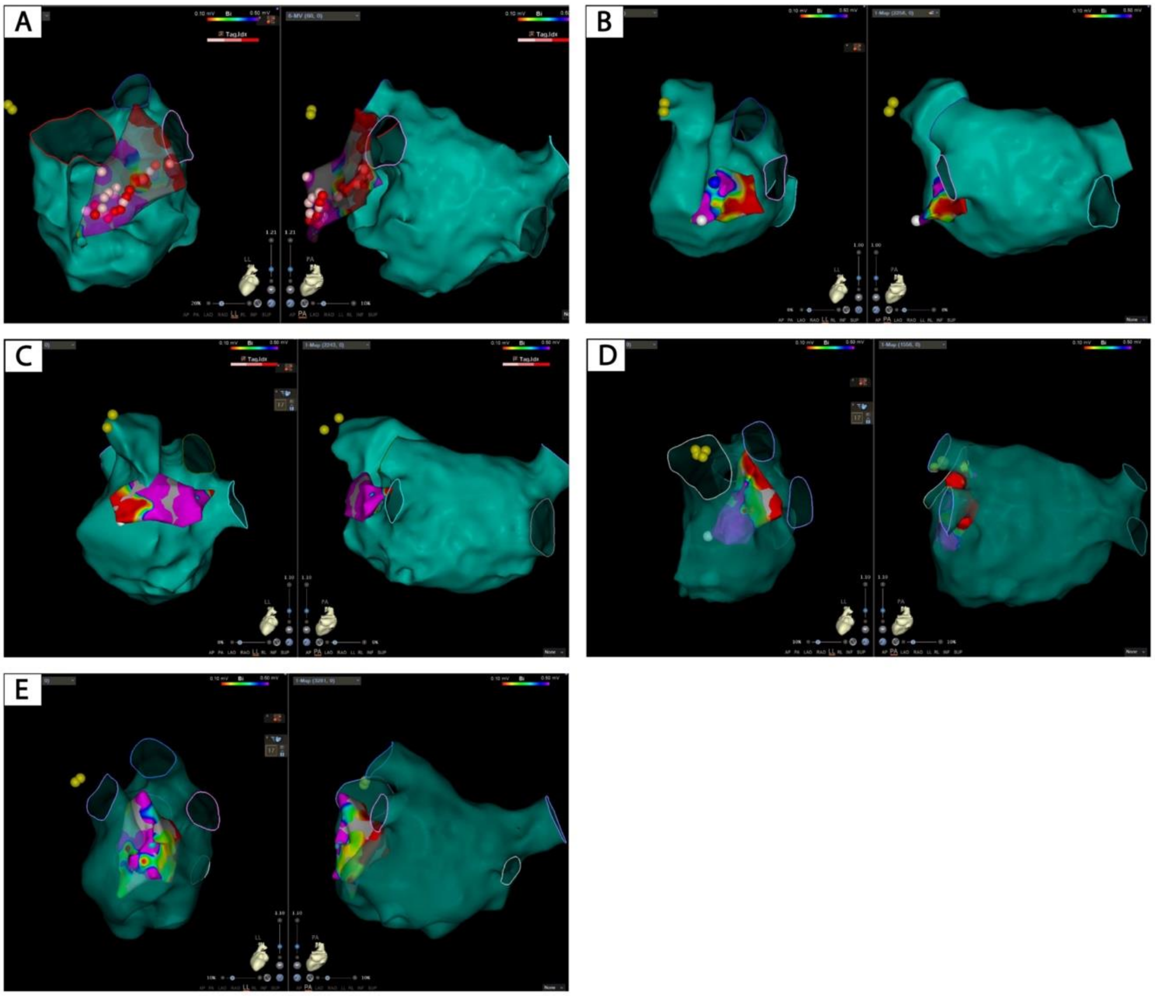

A-E: voltage maps of patients No. 3-7.
